# Monogenic Common Variable Immunodeficiency (Mo‐CVID) Score for Optimizing the Genetic Diagnosis in Pediatric CVID Cohort

**DOI:** 10.1002/eji.202451433

**Published:** 2025-03-13

**Authors:** Federica Barbati, Lorenzo Lodi, Silvia Boscia, Martina Cortimiglia, Elisa Calistri, Francesca Quaranta, Laura Maggi, Alessio Mazzoni, Boaz Palterer, Francesco Annunziato, Chiara Azzari, Silvia Ricci

**Affiliations:** ^1^ Pediatrics and Neonatology Unit Santo Stefano Hospital USL Toscana Centro Prato Italy; ^2^ Department of Neurofarba University of Florence Florence Italy; ^3^ Department of Health Sciences University of Florence Florence Italy; ^4^ Immunology Unit Meyer Children's Hospital IRCCS Florence Italy; ^5^ Department of Experimental and Clinical Medicine University of Florence Florence Italy; ^6^ Center of Flow Cytometry and Immunotherapy (CDCI) Careggi University Hospital Florence Italy; ^7^ Laboratory of Clinical Immunology and Microbiology National Institute of Allergy and Infectious Diseases National Institutes of Health Bethesda Maryland USA

**Keywords:** children, common variable immunodeficiency, exome sequencing, primary immunodeficiency, prioritization

## Abstract

Common variable immunodeficiency (CVID) represents an “umbrella” diagnosis due to its clinical and immunological heterogeneity. The primary objective of this study was to describe a cohort of CVID pediatric subjects from clinical, immunological, and genetic viewpoints. Secondary, we propose a model for prioritizing genetic investigations in these patients. Thirty‐four patients with CVID followed at Meyer Children's Hospital, IRCSS, were enrolled. Whole exome sequencing was performed according to the latest International Union of Immunological Societies 2022 update. Genetic variants were identified in 16 patients (47%), including known variants in SLC39A7, PRKCD, STAT3, NFKB1, PIK3R1, PLCG2, RFXANK, PRKDC, TNFRSF13B, and novel variants in SPI1, NFKB1, NFKB2. Comparing the Gene+ and Gene− cohorts, we demonstrated that a monogenic cause is more likely to be found in cases of early disease onset, positive family history, autoimmunity, lymphoproliferation, and specific immunological alterations. Using these criteria, we developed a pediatric monogenic CVID (Mo‐CVID) score to hypothesize when a CVID pediatric patient is more likely to carry a genetic mutation. A scoring system such as the Mo‐CVID score could help physicians prioritize genetic testing. Genetic analysis in CVID patients can help stratify patients into different disease entities to predict complications and prognosis, ensure appropriate genetic counseling, and personalize treatment.

## Graphical abstract

A mutation was identified in 47% of the CVID‐enrolled patients and, in the majority, it was not related to CVID according to the IUIS classification, leading to a diagnostic metamorphosis of this pathological entity. The Mo‐CVID Score could help clinicians prioritize genetic analysis in pediatric CVID patients. The image was created with BioRender.com.

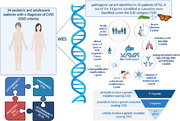

**Abbreviations**
CID: combined immunodeficiencies
CVID: common variable immunodeficiency
ESID: European Society for Immunodeficiencies
IEIs: inborn errors of immunity
IQR: interquartile range
IUIS: International Union of Immunological Societies
Mo‐CVID: monogenic CVID
NPV: negative predictive value
PPV: positive predictive value
ROC: receiver operating characteristic
SCID: severe combined immunodeficiencies
VUS: variants of uncertain significance
WES: whole exome sequencing

## 1 Introduction

Common variable immunodeficiency (CVID) is the most frequent symptomatic primary humoral immunodeficiency [1], affecting both children and adults worldwide with an estimated prevalence of 1:25,000–1:50,000 [2]. CVID has been variably defined over the years, due to its clinical and immunologic heterogeneity. It represents an “umbrella” definition of all those clinical forms characterized by increased susceptibility to infections, lymphoproliferation, autoimmune, autoinflammatory, or neoplastic manifestations [3]. According to the European Society for Immunodeficiencies (ESID) registry [4], laboratory criteria are low IgG and IgA or IgM and at least one between poor antibody vaccine response or low switched memory B cells. The diagnosis of CVID can be made after the 4th year of life (but symptoms may be present before) [4].

In the last decade, the spread of next‐generation sequencing has led to the identification of a growing number of inborn errors of immunity (IEIs) [5]. The last classification from the International Union of Immunological Societies (IUIS) expert committee on IEIs was published in 2022 and included 485 entities [6, 7].

Whole exome sequencing (WES) is presently considered the most cost‐effective approach for the discovery of genetic defects in patients with IEIs [5]; however, the results of its application in pediatric patients with CVID are not well known, as there are few specific pediatric studies published. The clinical and genetic heterogeneity of CVID and the resources required for sequencing have hindered the identification of the underlying genetic defect of the disease, allowing the identification of monogenic genetic variants from 2% to 30% of CVID patients in different cohorts [8, 9, 10, 11, 12]. The objective of this study is twofold: first, to describe a cohort of pediatric and adolescent patients with CVID and comprehensively characterize them from clinical, immunological, and genetic perspectives. Second, the study aims to develop a scoring system to aid clinicians in identifying pediatric patients with a high probability of CVID diagnosis, indicating the likelihood of finding a genetic mutation as unlikely, possible, or probable.

## 2 Methods

Patients followed at the Immunology Unit of Meyer Children's Hospital, IRCCS, Florence, Italy, with a diagnosis of CVID in accordance with the ESID criteria were recruited. Patients with a clinical/laboratory presentation of SCID/CID at the time of enrollment were not included.

Patient's clinical and laboratory data were retrospectively recorded from the medical electronic records. When available, samples from parents and siblings were submitted for whole‐exome to study familial segregation.

Genetic and flow cytometry and immunologic testing were carried out by the Immunology Laboratory, Meyer Children's Hospital, IRCSS, and by the Center of Flow Cytometry and Immunotherapy, Careggi University Hospital, Florence, Italy.

### 2.1 Flow Cytometry

All flow cytometric analyses were performed on fresh whole blood samples drawn in the previous 24 h in ethylenediaminetetraacetic acid to prevent coagulation and conserved at room temperature. A standard TBNK panel including CD45, CD3, CD4, CD8, HLA‐DR, CD19, CD16, and CD56, was used to analyze the main lymphocyte populations; T and B cells subpopulations analysis are reported in Supporting Information materials.

Samples acquisition was performed using FACS Lyric (BD Biosciences) and data were analyzed with the FACS Suite (BD Biosciences) software.

### 2.2 Genetic Analysis

Peripheral blood DNA was extracted using QIAamp Mini Kit (QIAGEN, Hilden, Germany) according to the manufacturer's instructions and quantified by Qubit 4 Fluorometer (Thermo Fisher Scientific).

According to their clinical phenotype and laboratory results, we performed for each patient the study of “clinical exome”: we started by analyzing the genetic panels listed in the IUIS classification that were most likely to include the mutation present in our patient and if we did not find the mutation, we expanded the search to include all the 485 genes known to cause immunodeficiencies and reported in the IUIS 2022 classification [7]. In case of negative genetic results, we then studied the whole exome.

Heterozygous and homozygous mutations were excluded if the allele frequencies in the general population were >5.0% in the GnomAD Exome database. Candidate mutations were manually confirmed by examining read alignment in the integrated genomics viewer. All variants with an “Alt Allele depth” of less than 45 were confirmed by Sanger Sequencing. Prediction scores were calculated using ClinVar [13] and AlamutVisualPlus and the variant pathogenicity was verified with Human Gene Mutation Database Professional [14]. The pathogenicity of all disease‐attributable gene variants was evaluated using the updated guideline for interpretation of molecular sequencing by the American College of Medical Genetics and Genomics considering the allele frequency, computational data, immunological/functional data, familial segregation and parental data, and clinical phenotyping. The classification of identified variants was performed according to the five‐tier scheme as recommended by the American College of Medical Genetics and Genomics [15] in “pathogenic”, “likely pathogenic”, “of uncertain significance” (VUS), “likely benign” and “benign”. Variants should be classified as VUS if other criteria are unmet or the criteria for benign and pathogenic are contradictory. VUS were then classified as hot, warm, tepid, cool, cold, and ice cold VUS. The details of the WES analysis are reported in Supporting Information materials.

### 2.3 Statistical Analysis

Data were processed using GraphPad Prism software version 9.5.1 (Dotmatics). Continuous variables were expressed as mean and standard deviation and median and interquartile range, when appropriate. We used Chi‐square or Fisher exact test for categorical variables, depending on the number of observations. Two‐sided *p* < 0.05 was considered statistically significant. Cut‐off values of the scoring system were determined using a receiver operating characteristic (ROC) curve.

### 2.4 Scoring System

To design the scoring system, we compared the occurrence of demographic, clinical, and laboratory features in gene‐positive and gene‐negative patient groups using the Chi‐square or Fisher's exact test. Based on these results, we selected 8 significant features, and we calculated the sensitivity, specificity, positive predictive value (PPV), negative predictive value (NPV), and accuracy for each characteristic. Then, we allocated a weighted score to each of them according to the significance of the *p*‐value (*p* 0.05–0.03 = 1 point, *p* 0.029–0.002 = 2 points, *p* ≤ 0.001 = 3 points), with the maximum clinical score being 13 points (when all features are present). The total clinical score was calculated for each patient. We determined cut‐off values of the total score to differentiate between likely and unlikely monogenic cases, based on a ROC curve.

### 2.5 Ethics

The study protocol was approved by the Regional Ethical Committee for Clinical Trials of Tuscany Region on August 18, 2020, and by the Regional Pediatric Ethical Committee of Tuscany Region on November 10, 2020. Written informed consent was obtained from the patients and minor(s)’ parents/legal guardians, for the genetic analysis and the publication of any data included in this study.

## 3 Results

A cohort of 34 pediatric and adolescent patients with a diagnosis of CVID was recruited; 12 (35.3%) were females and 22 (64.7%) were males. Twenty‐nine (85.3%) patients were Caucasian (27 Italian, 1 Ukrainian, and 1 Armenian), while the other 5 patients (14.7%) had different nationalities: 2 Tunisian, 1 Bosnian, 1 Indian, and 1 Moroccan. The parents of three patients (8.8%) were relatives, while the others 31 (91.2%) denied consanguinity.

Of the 34 patients, 22 (64.7%) were sporadic cases and 12 (35.3%) had a known family history of immunodeficiency. At the time of data analyses the average age of the patients was 14 ± 5.5 years, while the median age was 15.5 years (interquartile range (IQR) 10–18 years).

According to the ESID criteria, the diagnosis of CVID was made after the age of 4 years old, but when reviewing the medical records retrospectively, we found that the median age of CVID clinical manifestations onset was 3 years (IQR 5 months–6 years) while the median age of the hypogammaglobulinemia diagnosis was 4 years (IQR 3–9 years). An average time of 19.4 months (standard deviation ± 31.6 months) passed between the clinical onset and the diagnosis of hypogammaglobulinemia.

### 3.1 Clinical and Immunological Phenotype

According to the clinical manifestations, we divided the 34 patients into two groups: 16 patients (47.1%) had an “infections‐only” phenotype, while the other 18 patients (53%) had a “complicated” phenotype. We considered the “complicated” phenotype patients that in addition to infections had also any autoimmune, lymphoproliferative, or neoplastic diseases.

Eight (23.5%) patients had severe infections, while 11 (32.4%) had infections with sequalae.

Severe infections were defined according to previous studies [16, 17] and according to the “Common Terminology Criteria for Adverse Events” [18] as those that required intravenous treatment or hospitalization. Infections were considered complicated by sequalae for example in the case of eardrum perforation with consequent flat tympanogram, development of chronic cough, or broncho pneumopathy with bronchiectasis or obliterative bronchiolitis.

Of the 18 patients with the “complicated” phenotype, 10 patients (29.4%) had both autoimmunity and lymphoproliferation, 1 (2.9%) had only lymphoproliferation, and 7 (20.6%) had only autoimmune manifestations. Neoplastic manifestations were not detected.

Autoimmunity was reported in 17 patients (50%): the autoimmune involvement was of one organ in 9 patients (26.5%), of two systems in 3 patients (8.8%), and of three or more in 5 patients (14.7%). The most frequent autoimmune manifestations were cytopenia (autoimmune thrombocytopenia followed by hemolytic anemia), enteropathy, psoriasis, thyroiditis, and rheumatological manifestations including dermatomyositis and juvenile idiopathic arthritis.

Lymphoproliferation was detected in 11 patients (32.4%): 4 patients (11.8%) had only one organ involved, while 7 patients (20.6%) had two or more systems involved. The most recurrent lymphoproliferative manifestations were chronic lymphadenopathies and splenomegaly.

In addition to all these clinical manifestations typically associated with CVID, we found other symptoms involving different organs and systems. Since the presence of all these heterogeneous clinical manifestations, also the medical therapies were very varied.

Typical and atypical clinical manifestations are summarized in Table S1; the details of the first clinical and atypical manifestations, most frequent infections, and specific therapies are reported in Supporting Information materials.

The immunological phenotype of each patient was studied and classified according to the Freiburg [19], Paris [20], and EUROClass [21] classifications; then we compared the frequency of each subclass in the group of patients with “infections‐only” and the group with “complicated” but we did not find significant associations (Table S2). Flow cytometric data of all the patients are reported in Table S3.

### 3.2 WES Results

After sequence alignment and variant calling, 34 patient exomes were filtered to identify variants in the list of candidate immunodeficiency‐associated genes according to the IUIS 2022 classification. The variants in the gene panel were then filtered for rare (<5% from GnomAD), exonic or splicing, and nonsynonymous variants. The variants were then classified and manually filtered to identify pathogenic, likely pathogenic, and VUS coherent with disease inheritance (biallelic, monoallelic) and family history.

**FIGURE 1:**
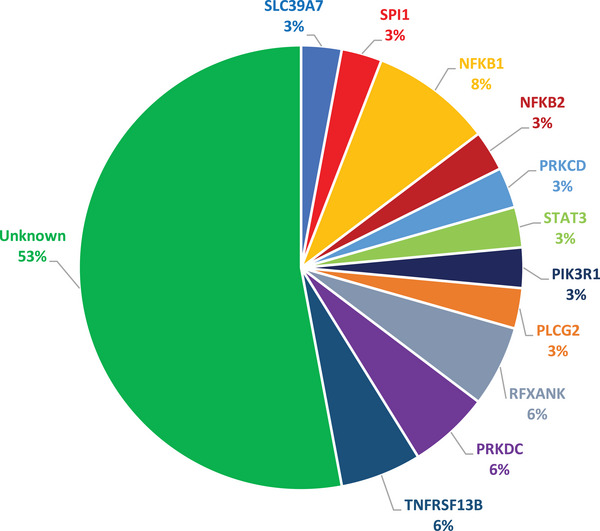
Percentage of patients in whom at least one candidate genetic variant was identified, divided into single genes and their overall frequency in the cohort: Whole exome sequencing was performed according to the latest International Union of Immunological Societies 2022 update in 34 pediatric patients with diagnosis of Common variable immunodeficiency. A putative pathogenic variant was identified in 16 patients of the 34 (47%) patients under study. We identified variants in 11 genes (SLC39A7, SPI1, NFKB1, NFKB2, PRKCD, STAT3, PIK3R1, PLCG2, RFXANK, PRKDC, TNFRSF13B).

A putative pathogenic variant was therefore identified in 16 patients (47%) (Figure 1; Table S4). We identified 14 variants in 10 genes (SLC39A7, SPI1, NFKB1, NFKB2, PRKCD, STAT3, PIK3R1, PLCG2, RFXANK, PRKDC), across 14 patients, 8 monoallelic variants, and 6 biallelic variants (3 homozygous and 3 compound heterozygous).

In five patients we found four variants of TNFRSF13B, but we considered as causative of the CVID phenotype only the mutations found in a compound heterozygous (in one patient) and homozygous (in another patient) state and not those in heterozygous form, in line with what is reported in the literature, as mentioned in the discussion.

All the variants were already reported in the literature, except for the variant found in the SPI1 gene, in the NFKB2 gene, and one of the two variants in NFKB1.

Four (TNFRSF13B, NFKB1, NFKB2, and PIK3R1) of these genes are included in the CVID phenotype IUIS table (Table 3.2, Severe reduction in at least two serum immunoglobulin isotypes with normal or low number of B cells, CVID phenotype), while the other seven are included in different IEIs tables [6]: RFXANK in TABLE 1.3 (combined immunodeficiencies (CID), generally less profound than severe CID (SCID)), PRKDC in Table 1.2 (T‐B‐SCID), SLC39A7 and SPI1 in Table 3.1 (severe reduction in all serum immunoglobulin isotypes with profoundly decreased or absent B cells, agammaglobulinemia), STAT3 gain‐of‐function in Table 4.3 (disease of immune dysregulation, regulatory T cell defects), PRKCD in Table 4.7 (disease of immune dysregulation, susceptibility to Epstein–Barr virus and lymphoproliferative conditions), and PLCG2 in Table 7.2 (autoinflammatory disorders, defects affecting the inflammasome). Clinical characteristics of patients carrying pathogenic/likely pathogenic variants are reported in Table S5.

### 3.3 Pediatric Mo‐CVID Score

Based on the results of the genetic analysis, patients were divided into two cohorts: gene‐positive (Gene+) with 16 patients and gene‐negative (Gene−) with 18 patients.

Then, we compared the occurrence of demographic, clinical, and laboratory features in the two groups; results are summarized in Table S6.

**FIGURE 2:**
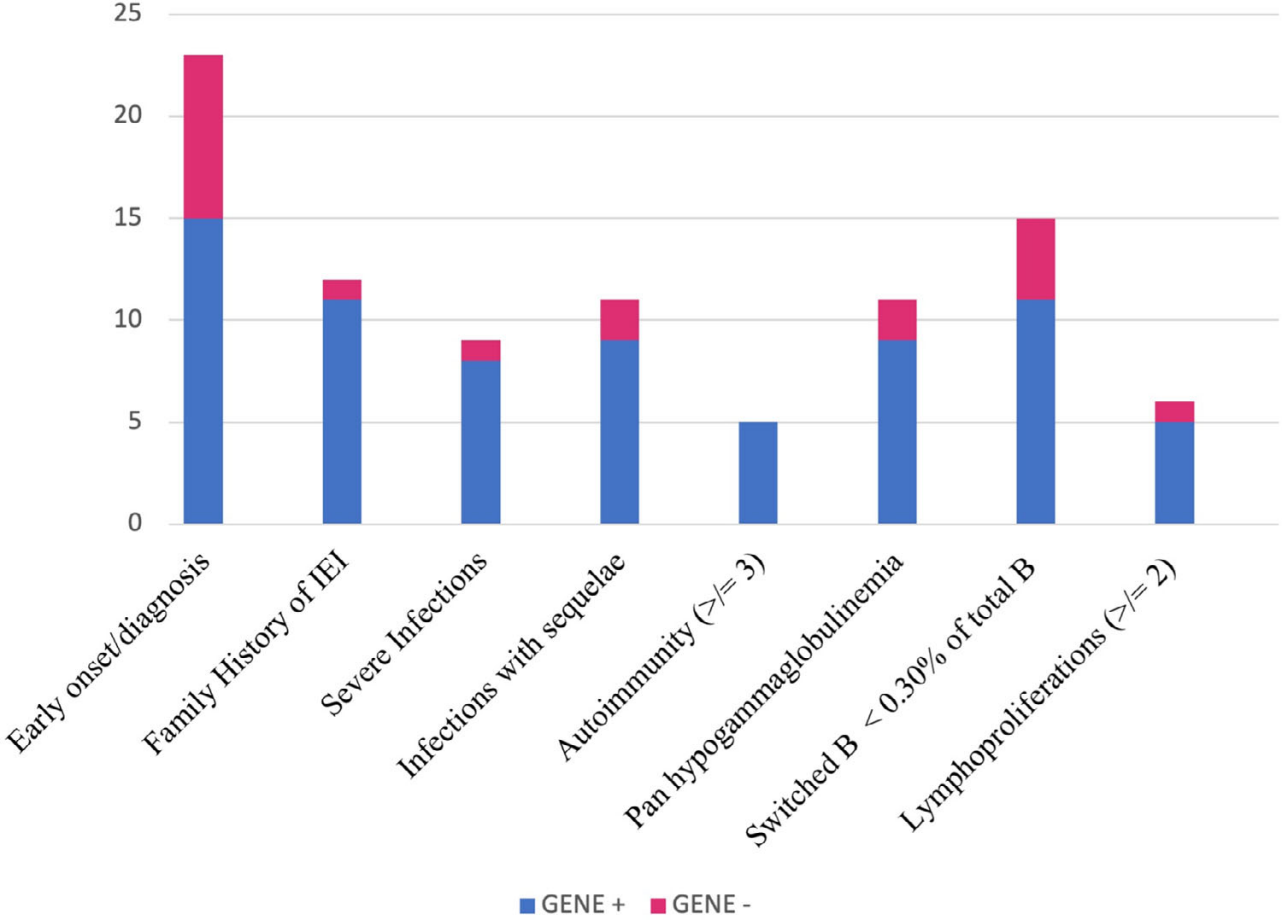
Bar chart giving an overview of the distribution of the eight significantly different (*p* < 0.05) features between Gene + and Gene− groups: Based on the results of the genetic analysis, patients were divided into two cohorts: gene‐positive (Gene +) with 16 patients and gene‐negative (Gene−) with 18 patients. Then, we compared the occurrence of demographic, clinical, and laboratory features in the two groups. We used Chi‐square or Fisher exact test for categorical variables, depending on the number of observations. Two‐sided *p* < 0.05 was considered statistically significant. The eight features are significantly different between Gene+ and Gene− groups were selected for the determination of the pediatric Mo‐CVID score: family history of IEIs, severe infections, infections with sequelae, pan hypogammaglobulinemia, absence of switched memory B cells, ≥3 autoimmune manifestations, ≥2 lymphoproliferative manifestations and clinical onset <4 years, or CVID diagnosis <7 years.

The eight features were significantly different (*p* < 0.05) between Gene+ and Gene− groups were selected for the determination of the pediatric Mo‐CVID score: family history of IEIs, severe infections, infections with sequelae, pan hypogammaglobulinemia (IgG, IgA, IgM deficiency), absence of switched memory B cells (<0.30%, 5 cells) of total B lymphocytes, ≥3 autoimmune manifestations, ≥2 lymphoproliferative manifestations, and clinical onset <4 years or CVID diagnosis <7 years (Figure 2).

Sensitivity, specificity, PPV, NPV, accuracy, and a weighted score based on the *p*‐value of each feature is reported in Table 1.

**TABLE 1:** Sensitivity, specificity, PPV, NPV, accuracy, *p*‐value, and assigned score of each feature of the pediatric Mo‐CVID score system.

Features	Sensitivity (%)	Specificity (%)	PPV (%)	NPV (%)	Accuracy (%)	*p‐*value	Score
Family history of IEIs	91.7	70	64.7	93.3	78.1	0.001	3
Severe Infections	88.9	60.9	47.1	93.3	68.8	0.018	2
Infections with sequelae	81.8	61.9	52.9	86.7	68.8	0.028	2
Pan hypogammaglobulinemia	81.8	61.9	52.9	86.7	68.8	0.028	2
Switched B < 0.30% of total B	73.3	64.7	64.7	35.3	68.8	0.042	1
Autoimmunity (≥3)	100	55.6	29.4	100	62.5	0.046	1
Lymphoproliferations (≥ 2)	83.3	53.8	29.4	70.6	59.4	0.048	1
Early onset/diagnosis^a^	65.2	77.8	88.2	46.7	68.8	0.049	1

Abbreviations: NPV, negative predictive value; NS, not significant; PPV: positive predictive value.

^a^Clinical onset < 4 years or CVID diagnosis < 7 years.

**FIGURE 3:**
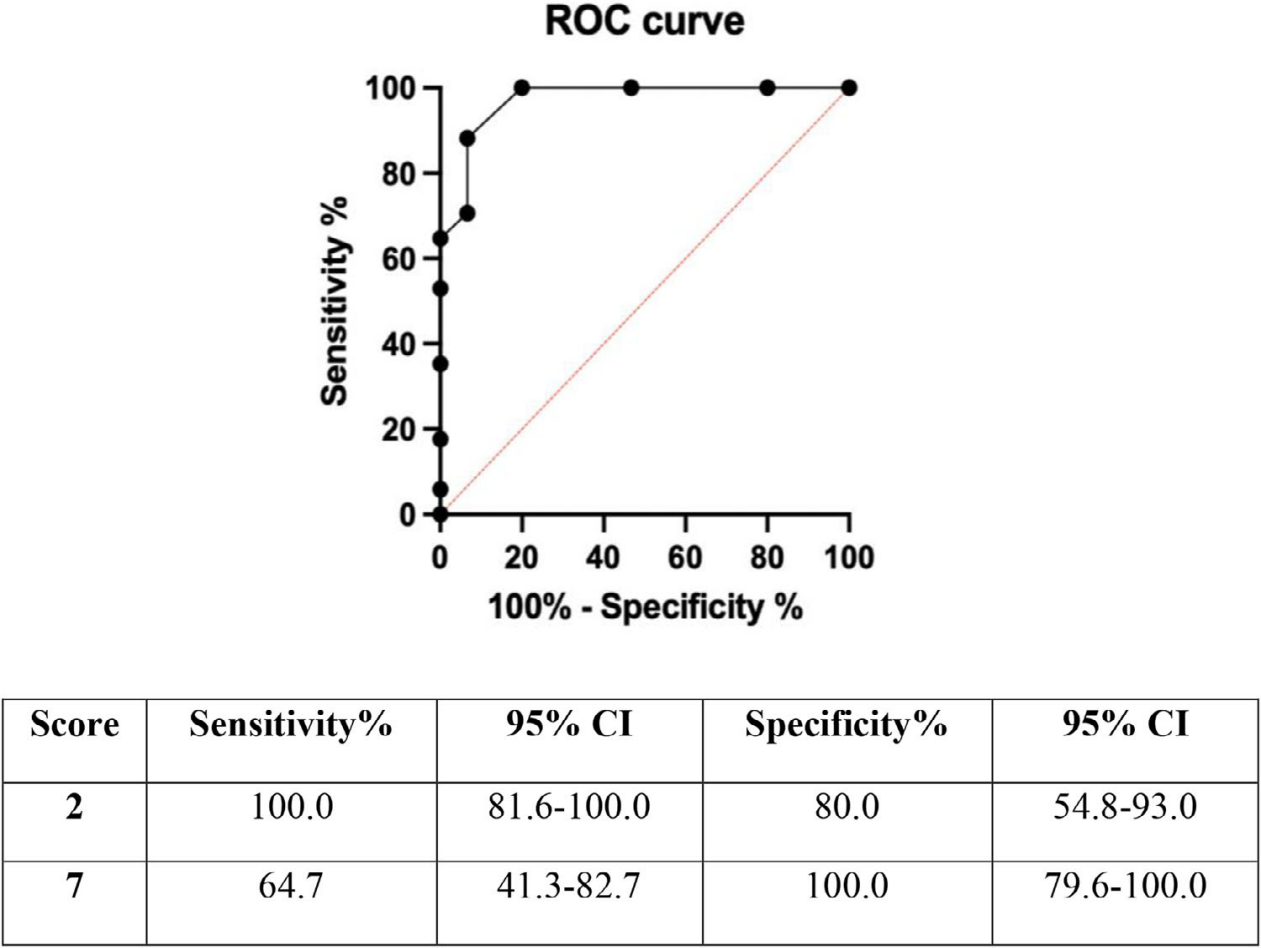
ROC curve and coordinates used for determination of relevant cut‐off scores to differentiate between likely and unlikely monogenic CVID cases: We identified 8 significantly different (*p* < 0.05) features between gene‐positive (Gene+, 16 patients) and gene‐negative (Gene−, 18 patients) groups and we calculated the sensitivity, specificity, positive predictive value, negative predictive value, accuracy and a weighted score for each characteristic. We determined cut‐off values of the total score to differentiate between likely and unlikely monogenic cases, based on a ROC curve. Cut‐off values of the scoring system were determined using a receiver operating characteristic (ROC) curve. Based on the sensitivity and the 1– specificity for each score, we determined a cut‐off for unlikely monogenic CVID at a score of 2 (sensitivity 100.0% [95% CI, 81.6−100.0]; specificity 80% [95% CI, 54.8−93.0]) and for likely monogenic CVID at a score of 7 (sensitivity 64.7% [95% CI, 41.3−82.7]; specificity 100.0% [95% CI, 79.6−100.0]).

The ROC curve and the coordinates used for the determination of relevant cut‐off scores to differentiate between likely and unlikely monogenic CVID cases are shown in Figure 3. Based on the sensitivity and the 1–specificity for each score, we determined a cut‐off for unlikely monogenic CVID at a score of 2 (sensitivity 100.0% [95% CI 81.6–100.0]; specificity 80% [95% CI 54.8–93.0]) and for likely monogenic CVID at a score of 7 (sensitivity 64.7% [95% CI 41.3–82.7]; specificity 100.0% [95% CI 79.6–100.0]).

**FIGURE 4:**
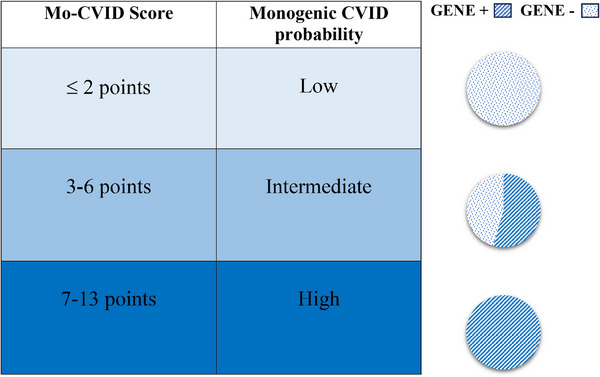
Pediatric Mo‐CVID Scoring system and graphical distribution of our Gene + and Gene− patients according to the score: To design the scoring system, we selected 8 significantly different features between gene‐positive (Gene +, 16 patients) and gene‐negative (Gene−, 18 patients) groups. Then we calculated the sensitivity, specificity, positive predictive value, negative predictive value, and accuracy for each characteristic and we allocated a weighted score to each of them according to the significance of *p*‐value (*p* 0.05–0.03 = 1 point, *p* 0.029–0.002 = 2 points, *p* ≤ 0.001 = 3 points), with the maximum clinical score being 13 points (when all features are present). The total clinical score was calculated for each patient and we determined cut‐off values of the total score to differentiate between likely and unlikely monogenic cases, based on an ROC curve. According to the Mo‐CVID score at ≤ 2 points, the subject is unlikely to have a genetic mutation causing CVID, at 3–6 points, the presence of a genetic cause is possible, and at 7–13 points, the subject is very likely to have a genetic mutation causing CVID. In our cohort, 13 (72.2%) of the Gene− and none of the Gene + group had a score ≤ 2 points; 5 (27.8%) of the Gene− and 5 (31.3%) of the Gene+ had a 3–6 points score; none of the Gene− and 11 (68.8%) of the Gene+ had a 7–13 points score.

Therefore, findings were consistent with the following total‐point score assignments: at ≤ 2 points, the subject was unlikely to have a genetic mutation causing CVID; at 3–6 points, the presence of a genetic cause was possible; and at 7–13 points, the subject was very likely (probable) to have a genetic mutation causing CVID (Figure 4).

In our cohort, 13 (72.2%) of the Gene− and none of the Gene+ group had a score of ≤2 points; 5 (27.8%) of the Gene− and 5 (31.3%) of the Gene+ had a 3–6 points score; none of the Gene− and 11 (68.8%) of the Gene+ had a 7–13 points score (Figure 4).

## 4 Discussion

Historically, the diagnosis of IEIs relied on clinical evaluation, family history, laboratory diagnostics, and immunophenotyping. However, the landscape has evolved with the integration of next‐generation sequencing into clinical practice. While hypogammaglobulinemia characterizes CVID and Ig replacement therapy is fundamental, pinpointing specific causative genes can unveil novel treatment targets. This advancement has revolutionized diagnostic and therapeutical approaches by enabling the identification of causative genetic mutations. In the case of CVID, accumulating data on genetic variants and their disease‐causing potential underscores the importance of genetic analysis. Nevertheless, the genetic underpinnings of CVID remain complex, necessitating further exploration of disease pathogenesis [22, 23]. In this study, we characterized from a clinical, immunological, and genetic point of view, a cohort of pediatric and adolescent patients meeting the ESID diagnostic criteria for CVID. In addition to the typical clinical manifestations related to CVID, in our cohort of patients several atypical symptoms involving different systems were detected, requiring multidisciplinary follow‐up and different treatment strategies. CVID, like all the other IEIs, are multisystemic complex diseases that can be characterized by a heterogeneous cohort of symptoms involving all organs and systems, and that therefore often require a multidisciplinary approach with the collaboration of several specialists.

As previously reported [24, 25], our results confirm that the most frequent clinical manifestations of CVID at onset are recurrent infection (in more than half of our patients), especially recurrent respiratory infections, followed by immune dysregulation (in almost a quarter of our patients). Atopy was detected in almost 18% of our patients, a percentage comparable to that observed in the healthy pediatric population [26]. However, due to the relatively small size of our cohort, further studies are needed to confirm this data also considering other IEIs.

The diagnostic delay in our cohort was similar to that reported for IEIs [27, 28], but lower considering only CVID patients, in particular adult cohorts [29, 30].

Although immunophenotyping plays an important role in the diagnosis and prognostic stratification of CVID patients, scarce data is available about immunophenotype‐genotype correlations. In our cohort, we could not identify any statistically significant difference in immunophenotype classifications (EUROclass, Freiburg, Paris) between different clinical phenotypes or between gene‐positive and gene‐negative patient groups, and neither a significant phenotype‐genotype correlation.

Performing a clinical WES in our cohort of patients, we identified pathogenic/likely pathogenic variants, VUS, or mutations associated with IEIs in almost half of the patients. Previously reported mutations in SLC39A7, PRKCD, STAT3, NFKB1, PIK3R1, PLCG2, RFXANK, PRKDC, TNFRSF13B genes, and new variants in SPI1, NFKB2, and NFKB1 were found in our cohort.

To enhance the diagnostic performance of our analysis, according to the bi‐annual revision of the IUIS expert committee on IEIs classification, we perform a periodic re‐analysis of negative WES analysis. In our cohort, the exome re‐analyses with the IUIS classification update led us to the identification of genetic mutations in two patients. Indeed, WES analyses of the patient with the mutation on the SPI1 gene were negative in 2020, while the re‐analysis in 2022 showed the mutation on the SPI1 gene that was not reported in the 2019 IUIS classification [7, 31]. Similarly, the identification of the SLC39A7 mutation was possible only after the 2019 IUIS update because the gene was not reported in the 2017 IUIS version [32]. To the best of our knowledge, this is the cohort in which the higher proportion of genetic diagnosis has been made compared with previous studies on genetic etiologies of CVID and these data confirm that WES is the best diagnostic genetic approach to pediatric CVID. Identification of the causative gene and its functional significance in patients with a CVID phenotype may significantly alter the management modalities from Ig replacement to hematopoietic stem cell transplantation or specific targeted therapy [33]. In our cohort of patients, genetic analysis led us to the identification of different target therapies for example Rapamune in the patient with mutation in PRKCD gene, Janus kinase‐inhibitor in the girl with STAT3 mutation, selective phosphoinositide 3‐kinase‐delta (PI3Kδ) inhibitor in the patient with PIK3R1 mutation, hematopoietic stem cell transplantation in patients with RFXANK mutations. Going to evaluate the individual mutations found surprised us, only four of the mutated genes identified in our cohort are reported in the CVID phenotype table of the IUIS 2022 classification. For the other patients, while meeting the ESID diagnostic criteria for CVID, we identified variants in seven genes included in different IEIs tables [7], for example, CID, SCID, agammaglobulinemia, disease of immune dysregulation, and autoinflammatory disorders. This discovery led us to consider a possible metamorphosis of CVID, which, once studied from a genetic perspective, actually reveals very different IEIs. Considering the patients with mutations in typical CVID‐genes we identified two patients with biallelic mutations (one compound heterozygous and one homozygous) in the transmembrane activator and calcium‐modulating cyclophilin ligand interactor (TACI, TNFRSF13B) that were considered causative of the CVID phenotype.

To date, there is no univocal position in the literature regarding mutations in TNFRSF13B.

Mutations on TNFRSF13B have been identified in about 8%–10% of CVID patients but are sometimes found also in healthy subjects who were not hypogammaglobulinemic [34, 35, 36], hence they are generally considered disease‐associated rather than pathogenic [37, 38, 39].

In our cohort, we identified three monoallelic mutations on TNFRSF13B in three patients (C104R in two patients, S144X in one patient), but we did not include them in the positive gene cohort considering what was reported by Salzer et al. [34] that stated that mutations C104R, A181E and S194X did not result in impaired TACI expression in the heterozygous state, while C104R or S144X homozygosity abrogates APRIL binding.

Interestingly, the three patients in our cohort with a monoallelic mutation on TNFRSF13B had a mild CVID phenotype characterized mainly by recurrent respiratory infections and IgG subclass deficiency, while their mothers with two different mutations (compound heterozygous) on the gene had a more complex phenotype, characterized by granulomatous form of CVID or severe hypogammaglobulinemia. This finding confirms that the loss of 1 functional allele might lead to a degree of haploinsufficiency, as previously reported [40].

Given the conflicting data reported in the literature, further studies about proteomic, transcriptomic, and epigenetic on TNFRSF13B would be necessary.

To date, there are no clinical or laboratory guidelines regarding the genetic workup of patients presenting with CVID and there is a lack of structured guidance on how to give priority to genetic tests within clinical genetic services. Recently, some authors have attempted to investigate whether the clinical complications in CVID patients might lead the physician to suspect the presence of a genetic defect, in one or more of the previously established causal genes [41].

Prioritization should be based on considerations of health needs, medical benefits, and costs; according to the accountability for reasonableness principles, one of the prioritization criteria for genetic testing is the “likelihood of disease” meaning the patient‐specific likelihood of being affected by the disease/genetic mutation tested for [42]. Therefore, we aimed to create a scoring system that could help clinicians prioritize the analysis in pediatric patients with CVID likelihood of being affected by a specific gene mutation. Comparing our gene‐positive and gene‐negative cohorts of patients, we demonstrated that a monogenic cause is more likely to be found in case of early disease onset (in infancy or early childhood), positive family history, presence of several autoimmune and lymphoproliferative manifestations, and specific immunological alterations (pan hypogammaglobulinemia and defect in switched B memory cells on total B cells). Using these clinical and laboratory criteria, we developed a pediatric Mo‐CVID scoring system to hypothesize when a patient is more likely or unlikely to have a genetic mutation causing CVID.

The Mo‐CVID score holds potential value not only in resource‐constrained developing countries but also in high and middle‐income nations, where it can aid in prioritizing clinical genetic testing and conserving both economic and human resources (in case of limited resources but also to avoid overwhelming the laboratories, or to make the best use of staff working hours). This scoring system could assist clinicians in identifying pediatric CVID patients for whom clinical WES is recommended sooner or less likely to yield a genetic mutation. Moreover, we posit that the Mo‐CVID score is crucial for determining candidates for further genetic testing. For instance, individuals with a high Mo‐CVID score (“probable”) but negative genetic analysis could benefit from more frequent exome re‐analysis in light of new developments in the literature over time or more comprehensive genetic testing methods such as mRNA sequencing, whole genome sequencing, or CGH array.

Nevertheless, patients with a low Mo‐CVID score should still be followed and investigated even if at different times compared with others, because disease‐causing genes with hypomorphic roles or with “redundant” functions could still be involved.

The evaluation of a patient with suspected IEIs is neither complete nor accurate without the inclusion of genetic testing. Therefore, we believe that genetic testing should be advocated for all patients suspected of IEIs while acknowledging that the timing for such testing may vary. In conclusion, our score can be used as a predictor of finding a causative mutation in patients and therefore of genetic testing diagnostic success; based on this prediction, prioritization can be applied in contexts where it is necessary.

## 5 Data Limitations and Perspectives

The main limitation of the study was the small number of patients included. Indeed, the study was conducted as part of a project with a limited timeframe and finances, which made it impossible to perform many more genetic analyses during that period. A multicentric study with a larger pediatric CVID cohort to further evaluate our score could be interesting and useful to demonstrate its feasibility and efficacy in different clinical settings.

## 6 Conclusion

In conclusion, genetic analysis in CVID patients is important to gain a better fundamental understanding of disease pathogenesis and to help stratify patients into disease entities to predict complications and prognosis, ensure appropriate genetic counseling, and possibly targeted treatment. Our study demonstrates that extended genetic analysis in patients with CVID led to a metamorphosis of this pathological entity, and the purpose of the Mo‐CVID score could help clinicians and immunology laboratories target genetic investigations with a higher level of awareness.

## Author Contributions

**Federica Barbati**: Data curation, formal analysis, investigation, visualization, writing‐original draft, and writing–review and editing. Lorenzo Lodi, **Alessio Mazzoni, and Boaz Palterer**: Data curation, validation, and review and editing. Silvia Boscia, Martina Cortimiglia, **Elisa Calistri, and Francesca Quaranta**: Formal analysis, investigation, and methodology. **Laura Maggi, Francesco Annunziato, and Chiara Azzari**: Conceptualization, project administration, supervision, validation, writing–review and editing, and funding acquisition. **Silvia Ricci**: Conceptualization, data curation, investigation, project administration, resources, supervision, visualization, writing–original draft, and writing–review and editing. All authors read and approved the final manuscript.

## Conflicts of Interest

The authors declare no conflicts of interest.

## Supporting information



Supporting Information

## Data Availability

The datasets generated during and/or analyzed during the current study are available from the corresponding author upon reasonable request.
