# Supplementary material for: Monogenic Common Variable Immunodeficiency (Mo‐CVID) Score for Optimizing the Genetic Diagnosis in Pediatric CVID Cohort
Source: Eur J Immunol. 2025 Mar 13;55(3):e202451433. doi: 10.1002/eji.202451433 (PMC11905875; doi:10.1002/eji.202451433)
Supplement: Supplementary file 1 — Supporting Information [file EJI-55-e202451433-s001.docx]

**Supplementary Data**

**Supplementary Methods**

**Flow cytometry**

A standard TBNK panel including CD45, CD3, CD4, CD8, HLA-DR, CD19, CD16 and CD56, was used to analyze the main lymphocyte populations. T cell differentiation was assessed by using CD45, CD3, CD4, CD8, CD45RA, CCR7, CD31, TCRγδ, which allow to recognize T CD4 and CD8 naïve (CCR7+CD45RA+), central memory (CCR7+CD45RA-), effector memory (CCR7-CD45RA-), terminal effector memory (CCR7-CD45RA+), recent thymic emigrants (CCR7+CD45RA+CD31+).

Analyses of B cell subpopulations was assessed by using CD45, CD19, CD20, IgM, IgD, CD38, CD27 and CD21. It enables the differentiation of B naïve (CD19+, CD27–), total memory B cells (CD27+, CD19+), marginal zone-like, plasmablasts, transitional, and CD21low B cells.

Memory B cells (CD19+, CD27+) were than divided in two main subclasses by correlating the expression of CD27 with the expression of immunoglobulin IgD: CD19+, CD27+, IgD+ (not-switched memory cells) and CD19+, CD27+, IgD– (switched memory cells).

**Genetic analysis**

For Exome Sequencing, a strategy based on enzymatic fragmentation to produce dsDNA fragments followed by End repair, A-tailing, adapter ligation, and library amplification was used. Libraries were hybridized with the protocol SeqCap EZ Exome v2 and sequenced with the platform NextSeq 500/550. Reads were aligned with the human reference hg38 genome using Burrows-Wheeler Aligner (BWA). The reads produced were mapped to the reference genome and were analyzed with the Integrative Genome Viewer (IGV), 2013, Broad Institute software. At the same time, the variant call for identification of nucleotide variants was performed using the Genome Analysis ToolKit (GATK) Unified Genotyper Module. Variant calling format (VCF) annotations were performed using ANNOVAR and VarAFT; all variants were annotated with dbSNP v150, GnomAD, KAVIAR, 1000 genomes, and COSMIC databases.

**Supplementary Results**

**Clinical phenotype**

The first clinical manifestations in our cohort of 34 CVID patients were infections in 19 patients (55.9%), especially respiratory infections, and immune dysregulation in 8 patients (23.5%), in particular autoimmunity in 7 patients (20.6%) and lymphoproliferation in one patient (2.9%). In 7 patients (20.6%), hypogammaglobulinemia was detected before the clinical onset during investigations made for different reasons (IEIs family history, growth slowdown, or comorbidity).

The most frequent infections were upper respiratory tract infections, presented by 30 patients (88.2%) and including bronchitis, laryngitis, pharyngotonsillitis and sinusitis, followed by pneumonia in 12 patients (35.3%) and otitis media in 10 (29.4%). The most common bacterial agents were *Streptococcus pneumoniae*, *Mycoplasma pneumoniae*, *Hemophilus influenzae*, *Streptococcus pyogenes*, *Klebsiella pneumoniae* and *Pseudomonas aeruginosa*. Gastrointestinal infections, due to Salmonella or *Yersinia enterocolitica*, were reported in 6 patients (17.6%), as also herpetic infections including Varicella Zoster virus (VZV), Epstein-Barr virus (EBV), and Herpes simplex virus (HSV).

A patient presented a *Streptococcus agalactiae* meningoencephalitis while another one a viral encephalitis and a sepsis due to multidrug-resistant *Streptococcus epidermidis*.

In addition to the typical CVID clinical manifestations, in our court we found other symptoms involving different organs and systems. Seven patients (20.6%) were under a neuropsychiatric follow-up for disorders ranging from mild such as insomnia or anxiety disorder to more serious such as Attention Deficit Hyperactivity Disorder (ADHD), learning disorders, and mental retardation. Six patients (17.6%) had atopy and in particular 3 had inhalants allergy (one of them had also vernal keratoconjunctivitis), two atopic dermatitis and one amoxicillin allergy. Other 5 patients (14.7%) had endocrinological pathologies: 2 had growth hormone (GH) deficiency, 2 had hypogonadotropic hypogonadism and one obesity with insulin resistance. Four patients (11.8%) had gastrointestinal disorders such as recurrent abdominal pains and gastroesophageal reflux. Three patients (8.8%) had nephrological disorders including tubule interstitial nephritis and uveitis (TINU), interstitial nephritis and tubulopathy, while two patients (5.9%) had hematological disorders including erythroid aplasia and thalassemia major.

Since the presence of all these heterogeneous clinical manifestations, also the medical therapies are very varied. Twenty-eight patients (82.4%) were on Ig replacement; of the others 6, 3 refused the therapy and 3 had mild Ig deficiency. Eighteen (52.9%) patients were treated also with other drugs including: tumor necrosis factor (TNF)-alpha inhibitors (Etanercept, Infliximab) for intestinal bowel disease (IBD) and juvenile idiopathic arthritis; steroids, Rituximab (anti-CD20), Mycophenolate mofetil, Fostamatinib for the cytopenia; hydroxychloroquine and Janus kinase (JAK) inhibitor therapy JAK for dermatomyositis; transfusions for thalassemia major and erythroid aplasia; GH hormone for GH deficiency; thyroid hormones for hypothyroidism; azithromycin as anti-inflammatory in chronic lung disease.

**Supplementary Tables**

| **Typical clinical**  **manifestations** | **n** | **%** | **Typical clinical manifestations** | **n** | **%** |
| --- | --- | --- | --- | --- | --- |
| Infections  URT infections  Pneumonia  Otitis media  Gastrointestinal infections  Herpetic infections  Bronchiolitis  Fungal infections  CNS infections  Osteomyelitis  Sepsis    Lymphoproliferation  Chronic lymphadenopathies  Splenomegaly  Hepatomegaly | 34  30  12  10  6  6  4  3  2  1  1  11  8  7  4 | 100  88.2  35.3  29.4  17.6  17.6  11.8  8.8  5.9  2.9  2.9  32.4  23.5  20.6  11.8 | Autoimmunity  Thrombocytopenia  Hemolytic anemia  Enteropathy  Psoriasis  Autoimmune Thyroiditis  Rheumatological disease  Interstitial lung disease  Neutropenia  Hepatitis  Alopecia   Neoplastic disease | 17  10  6  3  3  3  3  3  1  1  1  0 | 50  29.4  17.6  8.8  8.8  8.8  8.8  8.8  2.9  2.9  2.9  0 |
| **Other clinical**  **manifestations** | **n** | **%** |  |  |  |
| Neuropsychiatric disorders  Atopy  Endocrinological disorders  Gastrointestinal disorders  Nephrological disorders  Hematological disorders | 7  6  5  4  3  2 | 20.6  17.6  14.7  11.8  8.8  5.9 |  |  |  |

**Table S1.** Clinical manifestations of the study CVID cohort. CNS: central nervous system; GH: growth hormone; TINU: tubule interstitial nephritis and uveitis; URT: upper respiratory tract.

| **EUROclass** | **Total, n (%)** | **Infections only, n** | **Complicated phenotype, n** | **p** |
| --- | --- | --- | --- | --- |
| B-  B+ SmB- Trhigh CD21lo B+ SmB- Trhigh CD21norm  B+ SmB- Trnorm CD21lo  B+ SmB- Trnorm CD21norm  B+ SmB+ CD21lo  B+ SmB+ CD21norm | 3 (8.8)  1 (2.9)  4 (11.8)  3 (8.8)  8 (23.5)  1 (2.9)  14 (41.2) | 2  0  2  0  5  0  7 | 1  1  2  3  3  1  7 | 0.591  1.000  1.000  0.230  0.429  1.000  1.000 |
| **Freiburg** | **Total, n (%)** | **Infections only, n** | **Complicated phenotype, n** | **p** |
| Ia  Ib  II | 1 (2.9)  16 (47)  17 (50) | 0  6  10 | 1  10  7 | 1.000  0.327  0.303 |
| **Paris** | **Total, n (%)** | **Infections only, n** | **Complicated phenotype, n** | **p** |
| MB0  MB1  MB2 | 23 (67.6)  9 (26.5)  2 (5.9) | 10  4  2 | 13  5  0 | 0.717  1.000  0.214 |

**Table S2.** Immunological phenotype of the study CVID cohort.

| **Pt** | **CD3+**  **(cells/μL)** | **CD3+CD4+**  **(cells/μL)** | **CD3+CD8+**  **(cells/μL)** | **CD4/CD8** | **CD16+CD56+**  **(cells/μL)** | **CD19+**  **(cells/μL)** | **CD19+CD27+**  **(%)** | **IgD-CD27+**  **(%)** | **CD21low**  **(%)** |
| --- | --- | --- | --- | --- | --- | --- | --- | --- | --- |
| **1*** | 4500 | 3150 | 1030 | 3,06 | 60 | 0 | 0 | 0 | 0 |
| **2*** | 1056 | 573 | 427 | 1,34 | 54 | 0 | 0 | 0 | 0 |
| **3*** | 1015 | 568 | 393 | 1,45 | 49 | 154 | 9 | 10 | 18.8 |
| **4*** | 1332 | 917 | 369 | 2,49 | 242 | 486 | 9 | 35 | 5.1 |
| **5*** | 1295 | 888 | 368 | 2,41 | 49 | 56 | 3 | 9 | 10.6 |
| **6*** | 1485 | 1035 | 407 | 2,54 | 163 | 231 | 2 | 50 | 2.8 |
| **7*** | 945 | 546 | 344 | 1,59 | 125 | 74 | 20 | 13 | 12.3 |
| **8*** | 1830 | 1145 | 455 | 2,52 | 46 | 13 | 0 | 0 | 0 |
| **9*** | 1386 | 703 | 561 | 1,25 | 287 | 522 | 16 | 38 | 2.0 |
| **10*** | 2405 | 695 | 1474 | 0,47 | 109 | 460 | 5 | 19 | 4.0 |
| **11*** | 1187 | 534 | 545 | 0,98 | 54 | 150 | 5 | 30 | 31.4 |
| **12*** | 1461 | 785 | 587 | 1,34 | 144 | 335 | 1 | 65 | 4.0 |
| **13*** | 1234 | 620 | 454 | 1,37 | 101 | 289 | 2 | 59 | 2.5 |
| **14*** | 1682 | 1018 | 542 | 1,88 | 169 | 543 | 8 | 19 | 7.0 |
| **15*** | 1583 | 719 | 715 | 1,00 | 439 | 670 | 14 | 31 | 3.1 |
| **16*** | 1012 | 482 | 347 | 1,39 | 83 | 287 | 42 | 36 | 3.2 |
| 17 | 2801 | 1418 | 1163 | 1,22 | 273 | 1196 | 9 | 22 | 2.8 |
| 18 | 994 | 564 | 394 | 1,43 | 136 | 60 | 24 | 49 | 3.5 |
| 19 | 1635 | 871 | 338 | 2,58 | 426 | 765 | 7 | 49 | 2.6 |
| 20 | 979 | 565 | 364 | 1,55 | 44 | 282 | 6 | 42 | 2.3 |
| 21 | 1840 | 1337 | 441 | 3,03 | 77 | 245 | 29 | 14 | 3.5 |
| 22 | 1562 | 1002 | 494 | 2,03 | 182 | 349 | 17 | 35 | 5.1 |
| 23 | 1359 | 721 | 544 | 1,33 | 183 | 175 | 29 | 26 | 6.0 |
| 24 | 1030 | 652 | 355 | 1,84 | 287 | 200 | 3 | 18 | 15.9 |
| 25 | 1866 | 892 | 734 | 1,22 | 140 | 473 | 19 | 30 | 4.9 |
| 26 | 1752 | 831 | 799 | 1,04 | 106 | 512 | 3 | 39 | 0.8 |
| 27 | 1467 | 887 | 387 | 2,29 | 233 | 389 | 13 | 64 | 1.9 |
| 28 | 1421 | 751 | 447 | 1,68 | 158 | 349 | 37 | 13 | 8.0 |
| 29 | 1001 | 577 | 331 | 1,74 | 135 | 165 | 8 | 17 | 2.4 |
| 30 | 1252 | 788 | 359 | 2,19 | 153 | 442 | 8 | 30 | 0.8 |
| 31 | 984 | 576 | 369 | 1,56 | 464 | 171 | 10 | 50 | 2.8 |
| 32 | 996 | 584 | 330 | 1,77 | 65 | 187 | 4 | 15 | 6.5 |
| 33 | 1066 | 529 | 386 | 1,37 | 90 | 230 | 10 | 20 | 3.6 |
| 34 | 1377 | 779 | 542 | 1,44 | 139 | 554 | 5 | 29 | 0.8 |

**Table S3.** Flow cytometric data of the 34 study CVID cohort. The reported values are the most significantly altered ones found in the patients. *16 patients of which the genetic analyses pointed out a causative genetic mutation.

| **Pt** | **Ref_gene** | **Ref_trans** | **Exon** | **Coding_seq** | **Prot_seq** | **Genotype** | **Molecular Consequence** | **dbSNP** | **ClinVar** | **Alamut/**  **ACMG** | **HGMD** |
| --- | --- | --- | --- | --- | --- | --- | --- | --- | --- | --- | --- |
| **1** | **SLC39A7** | NM_006979.3 | 6  6 | c.1126 A>C  c.849_850delAA | p.Ser376Arg  p.Val286fs*21 | Het #  Het # | Missense  Frameshift | /  rs780681928 | Uncertain Significance  Uncertain Significance | Hot VUS (score 5)  Pathogenic  (score 10) | /  / |
| **2** | **SPI1** | NM_001080547.2 | 5 | c.670 A>G | p.Met224Val | Het | Missense | / | / | Hot VUS (score 5) | / |
| **3**  **4**  **5** | **NFKB1**  **NFKB1**  **NFKB1** | NM_003998.4 | 12  10  10 | c.1189_1196delCACTGG  c.904dupT  c.850 C>T | p.Gly397Lysfs*7  p.Ser302Phefs*7  p.Arg284* | Het  Het  Het | Frameshift  Frameshift  Nonsense | /  rs773694113  rs1578793312 | /  Pathogenic/Likely pathogenic  Pathogenic/Likely pathogenic | Pathogenic  (score 10)  Pathogenic  (score 11)  Pathogenic  (score 11) | /  DM  DM |
| **6** | **PRKCD** | NM_006254.4 | Intron14 | c.1352+1 G>A | / | Hom | Splice donor | rs398122958 | Pathogenic | Pathogenic  (score 11) | DM |
| **7** | **STAT3** | NM_139276.3 | 9 | c.833 G>A | p.Arg278His | Het | Missense | rs2082128828 | Likely pathogenic | Likely pathogenic (score 6) | DM |
| **8** | **PIK3R1** | NM_181523.3 | Intron 11 | c.1425+1 G>A | / | Het | Splice donor | rs587777709 | Pathogenic | Pathogenic  (score 11) | DM |
| **9** | **PLCG2** | NM_002661.5 | 11 | c.923 C>T | p.Ala308Val | Het | Missense | rs199636472 | Conflicting interpretations of pathogenicity | Cold VUS (score 1) | DM? |
| **10**  **11** | **RFXANK**  **RFXANK** | NM_003721.4 | 6  6 | c.338- 25_338del26  c.338- 25_338del26 | p.Gly113Valfs*91  p.Gly113Valfs*91 | Hom  Hom | Splice acceptor  Splice acceptor | /  / | Pathogenic  Pathogenic | Pathogenic  (score 11)  Pathogenic  (score 11) | DM  DM |
| **12**  **13** | **PRKDC**  **PRKDC** | NM_006904.7 | 31  57  31  57 | c.3820 A>G  c.7649 T>C  c.3820 A>G  c.7649 T>C | p.Arg1274Gly  p.Ile2550Thr  p.Arg1274Gly  p.Ile2550Thr | Het #  Het #  Het #  Het # | Missense  Missense  Missense  Missense | rs559349334  rs373258150  rs559349334  rs373258150 | Uncertain Significance  Uncertain  Significance  Uncertain Significance  Uncertain Significance | Tepid VUS (score 3)  Tepid VUS (score 3)  Tepid VUS (score 3)  Tepid VUS (score 3) | /  /  /  / |
| **14** | **NFKB2** | NM_001077494.3 | 13 | c.1214 C>A | p.Ala405Glu | Het | Missense | rs773329863 | / | Tepid VUS (score 3) | / |
| **15**  **16** | **TNFRSF13B**  **TNFRSF13B** | NM_012452.3 | 3  3  4 | c.310 T>C  c.204dupA  c.579 C>A | p.Cys104Arg  p.Leu69Thrfs*12  p.Cys193* | Hom  Het #  Het # | Missense  Frameshift  Nonsense | rs34557412  rs72553875  rs72553885 | Conflicting interpretations of pathogenicity  Likely pathogenic  Pathogenic | Tepid VUS (score 3)  Likely pathogenic (score 8)  Pathogenic  (score 11) | DM  DM  DM |

**Table S4.** Selected rare candidate mutations identified in genes from the PID-associated gene panel in the 16 patients with a positive genetic analysis. ACMG: American College of Medical Genetics and Genomics; DM: disease causing mutation; DM?: disease causing mutation?; DFP: disease-associated polymorphism with supporting functional evidence; HGMD: Human Gene Mutation Database; VUS: variant of uncertain significance; #: Compound heterozygous.

| **Pt ID** | **Sex** | **Nationality** | **Age at CVID suspect**  **(years)** | **Mutated Gene** | **Infectious manifestations** | **Autoimmunity** | **Lymphoproliferation** | **Other clinical manifestation** | **Therapy** |
| --- | --- | --- | --- | --- | --- | --- | --- | --- | --- |
| 1 | M | Moroccan | 3,5 months | SLC39A7 | Streptococcus agalactiae sepsis and meningoencephalitis, oral and inguinal candidiasis | **/** | **/** | Eczematous dermatitis | Ig replacement |
| 2 | F | Italian | 3 | SPI1 | Chronic rhinosinusitis | ITP, thyroiditis | Hepatomegaly with hepatic fibrosis | Pulmonary dysventilation and fibrosis, erythroid aplasia | Ig replacement, blood transfusions, iodine supplements |
| 3 | F | Italian | 6 | NFKB1 | Bronchitis, pharyngotonsillitis, salmonella gastroenteritis, Herpes Zoster infection | Mild ITP | Hypermetabolic lymphadenopathy and mild splenomegaly | Bronchiectasis, papulopustular rosacea | Ig replacement |
| 4 | M | Italian | 2 | NFKB1 | Otitis, pneumonia, perianal abscess, Herpes Zoster infection | **/** | **/** | GH deficiency | Previous:GH replacement |
| 5 | M | Italian | 11 | NFKB1 | Otitis | ITP, haemolytic anemia | Hepatomegaly, Splenomegaly, abdominal lymphadenopathy | Interstitial lung disease, delayed stature growth | Ig replacement  Sirolimus,  antimicrobial prophylaxis |
| 6 | M | Bosnian | 10 | PRKCD | Otitis with eardrum perforation, Mycoplasma pneumoniae and RSV pneumonia, HHV6 encephalitis, Staphylococcus epidermidis sepsis | Trilineage cytopenia, hepatitis and enteritis | Splenomegaly, abdominal lymphadenopathy | Interstitial lung disease, delayed stature growth, alopecia areata | Ig replacement, Sirolimus, antimicrobial prophylaxis |
| 7 | F | Italian | 7 | STAT3 | Pneumonia, Yersinia gastroenteritis, Herpes Zoster infection | ITP, haemolytic anemia, thyroiditis | Hepatosplenomegaly, lymphadenopathy | Interstitial lung disease, colitis with lymphoid nodular hyperplasia | Ig replacement, prophylaxis with azithromycin, Ruxolitinib |
| 8 | F | Italian | 10 | PIK3R1 | Recurrent poxvirus skin infections | **/** | **/** | GH deficiency, specific learning disorder | Ig replacement, Leniolisib  Previous:GH replacement |
| 9 | M | Italian | 5 | PLCG2 | URTI with bronchospasm, otitis, laryngitis, chronic rhinosinusitis, gastroenteritis | **/** | **/** | Oral aphthosis, IBS | Ig replacement |
| 10 | M | Tunisian | 4 | RFXANK | Bronchitis, pneumoniae, oral candidiasis | Dermatomyositis | **/** | Mild keratoconjunctivitis, inhalant allergy | Ig replacement, antimicrobial prophylaxis, Baricitinib  Scheduled:  HSCT |
| 11 | M | Tunisian | 4 | RFXANK | Bronchitis, pneumoniae, otitis with eardrum perforation, salmonella gastroenteritis | **/** | **/** | Delayed stature growth, psoriasis, Vernal keratoconjunctivitis, recurrent episodes of diarrhea and vomiting, amoxicillin allergy | Ig replacement, antimicrobial prophylaxis  Scheduled:  HSCT |
| 12 | F | Italian | 1 | PRKDC | Bronchiolitis, pharyngitis, otitis with eardrum perforation, gastroenteritis | **/** | Lymphadenopathy | Oral aphthosis, bowel follicular lymphoid hyperplasia, reflux esophagitis | Ig replacement |
| 13 | F | Italian | 3 | PRKDC | Recurrent pneumonia, chronic sinusopathy | **/** | **/** | **/** | Ig replacement |
| 14 | F | Italian | 2 | NFKB2 | Otitis, bilateral otomastoiditis, chickenpox infection | **/** | **/** | Atopic dermatitis, speech delay | Ig replacement |
| 15 | M | Italian | 3 | TNFRSF13B | URTI, urinary tract infection, nail infection with granuloma | Urticaria-vasculitis | **/** | Oral aphthosis, conjunctivitis, atopic dermatitis | Ig replacement |
| 16 | M | Italian | 6 | TNFRSF13B | Herpetic stomatitis | Recurrent ITP | Abdominal lymphadenopathy | **/** | Ig replacement, MMF  Previous:  Eltrombopag |

**Table S5.** Clinical characteristics of the 16 patients carrying pathogenic/likely pathogenic variants related to IEIs.

HSCT: hematopoietic stem cell transplant; IBD: intestinal bowel disease; IBS: irritable bowel syndrome; Ig: immunoglobulin; ITP: immune thrombocytopenic purpura; GH: growth hormone; HHV6: Human Herpesvirus 6; MMF: Mycophenolate Mofetil; RSV: respiratory syncytial virus; URTI: upper respiratory tract infections.

| **Clinical features** | **p** | **Laboratory features** | **p** |
| --- | --- | --- | --- |
| **Autoimmunity (≥ 3)**  Autoimmune cytopenia  Bronchiectasis  Chronic diarrhea  Chronic lymphadenopathies  **Early onset/diagnosis***  Enteropathy  Hepatomegaly  **Infections with sequelae**  **Lymphoproliferations (≥ 2)**  Respiratory infections  **Severe Infections**  Splenomegaly | **0.046**  NS  NS  NS  NS  **0.049**  NS  NS  **0.028**  **0.048**  NS  **0.018**  NS | CD21low cells  EUROclass  Freiburg classification  **Pan hypogammaglobulinemia**  Paris classification  **Switched memory B cells < 0.30% of total B lymphocytes**  Two classes hypogammaglobulinemia | NS  NS  NS  **0.028**  NS  **0.042**  NS |
|  |  | **Demographic features** | **p** |
|  |  | Consanguinity  **Family history of IEIs**  Non-Caucasian ethnicity | NS  **0.001**  NS |

**Table S6.** Comparison between the occurrence of demographic, clinical and laboratory features in the gene-positive and the gene-negative patient groups. NS: not significant. *Clinical onset < 4 years or CVID diagnosis < 7 years.
